# Genetic and clinical characteristics in Japanese hereditary breast and ovarian cancer: first report after establishment of HBOC registration system in Japan

**DOI:** 10.1038/s10038-017-0355-1

**Published:** 2017-11-08

**Authors:** Masami Arai, Shiro Yokoyama, Chie Watanabe, Reiko Yoshida, Mizuho Kita, Megumi Okawa, Akihiro Sakurai, Masayuki Sekine, Junko Yotsumoto, Hiroyuki Nomura, Yoshinori Akama, Mayuko Inuzuka, Tadashi Nomizu, Takayuki Enomoto, Seigo Nakamura

**Affiliations:** 1 Clinical Genetic Oncology, Cancer Institute Hospital of Japanese Foundation for Cancer Research, Tokyo, Japan; 2 Breast Center, Showa University, Tokyo, Japan; 3 Faculty of Human Science, Sophia University, Tokyo, Japan; 4 St Luke’s International Hospital, Tokyo, Japan; 5 Department of Medical Genetics, Sapporo Medical University, Hokkaido, Japan; 6 Department of Obstetrics and Gynecology, Niigata University Graduate School of Medical and Dental Science, Niigata, Japan; 7 Natural Science Division, Faculty of Core Research, Ochanomizu University, Tokyo, Japan; 8 Department of Obstetrics and Gynecology, Keio University School of Medicine, Tokyo, Japan; 9 Clinic of Familial Tumor, Hoshi General Hospital, Fukushima, Japan; 10 Department of Surgery, Hoshi General Hospital, Fukushima, Japan

**Keywords:** Cancer epigenetics, Breast cancer

## Abstract

The hereditary breast and ovarian cancer (HBOC) registration system of Japan was established by the Japanese HBOC Consortium. The first trial was registered in 2015 in four institutions to which some registration committee members belonged. We analyzed the information of 830 Japanese pedigrees, who underwent *BRCA1/2* genetic testing, including mutation carriers with *BRCA1* (*N* = 127) and *BRCA2* (*N* = 115), and their families. The mutation-positive rate was 19.7%. Variants of uncertain significance were found in 6.5% of all individuals subjected to genetic testing for *BRCA1/2*. Compared to the United States, Japan had a higher mutation-positive rate in most categories, except for the groups with male breast cancer. Among the intrinsic subtypes of *BRCA1*-associated breast cancers, 75.8% were triple-negative. The incidence rate of contralateral breast cancer in *BRCA1/2* mutation carriers was 0.99%/year. Among 240 mutation carriers, 26 and 62 patients underwent risk-reducing mastectomy (RRM) and risk-reducing salpingo-oophorectomy (RRSO), respectively; the respective frequencies of occult cancer were 7.1 and 3.2%. Metachronous breast cancer after RRM or peritoneal cancer after RRSO was not observed during the follow-up period. The nationwide registration system began last year and the system enables follow-up analysis in Japan.

## Introduction

Hereditary breast and ovarian cancer syndrome (HBOC) is an autosomal dominant inherited cancer susceptibility disorder caused by deleterious germline mutations in *BRCA1* or *BRCA2* (*BRCA1/2*). Starting in 2004, *BRCA1/2* genetic testing became a part of laboratory examinations in Japan. However, medical insurance does not currently cover the clinical practice of HBOC, including genetic testing for *BRCA1/2*, medical surveillance, and preventive surgeries. Therefore, these procedures are not prevalent in routine clinical practice, and the clinical and pathological characteristics of HBOC in Japan remain unclear.

International database systems such as CIMBA (the Consortium of Investigators of Modifiers of BRCA1/2) or Asian BRCA consortium about BRCA1/2 have already been established [1, 2]. However, data from Japanese HBOC families are poorly represented in these international databases or collaborative studies. Comparison of genetic and clinical features between other foreign countries and Japan cannot be performed in detail. The Korean Hereditary Breast Cancer study (KOHBRA) characterized clinical and genetic features [3]. Therefore, the Japanese nationwide registration system specific to HBOC is definitely required to clarify clinical and genetic features of Japanese HBOC and to improve medical treatment of HBOC.

The Japanese HBOC Consortium (JHC) was established in December 2012, with 342 members in December 2016. The aim of JHC is to spread awareness of HBOC and to provide effective healthcare for patients with HBOC and their families [4]. We established a registration committee for JHC in October 2013, and pushed it forward as a nationwide registration project. Before formal nationwide registration, we first performed trial registration in four institutions, which, in total, include over 40% of cases with *BRCA1/2* genetic testing, to confirm that the electronic registration system and analytical system worked appropriately. Here, we report the results and issues of this registration system in JHC.

## Materials and Methods

### Subjects and registration

The registered subjects were all Japanese individuals who underwent *BRCA1/2* genetic testing (including individuals in which no mutation was detected) from January 2012 to December 2014. We began the trial registration at the following four medical institutions, to which some of the registration committee members belonged: St. Luke’s International Hospital (Tokyo), Cancer Institute Hospital (Tokyo), Showa University Hospital (Tokyo), and Hoshi General Hospital (Fukushima).

This study was approved by the Ethical Review Board of JHC in December 2014. After further approval by the Ethical Review Board or Institutional Review Board of each medical institution, we began this registration project. Informed consent was obtained from all subjects. If informed consent could not be received face to face for retrospective cases for whom medical treatment had been terminated or for patients who died, the candidate or the candidate’s family members could opt out on the homepages of JHC and each participating institute. All subjects received genetic counseling and genetic testing of their own free will in clinical practice, including recommendation for further evaluation by the doctor in charge. All genetic testing with sequence and large rearrangement analysis was performed at Myriad Genetic Laboratories or FALCO Biosystems. The detected variants were interpreted according to the criteria of Myriad Genetic Laboratories. Sequencing and large rearrangement analysis were ordered in all but 10 cases. Among them, only sequencing was performed even if pathogenic mutations were not detected by sequencing.

For each subject, we also collected data on history of any cancers within second-degree relatives and in cousins. We entered information for *BRCA1/2* genetic testing and the clinical and pathological findings of breast cancer, ovarian cancer, and other cancers in the original electronic template, which was referred to the previous Japanese research (Supplementary Fig. 1; Table 1) [5]. All data except sex were anonymously registered in each institution. The date of birth only included the year and month. The date was registered as 15 in all cases. Input batch data were sent to the datacenter together, which is located in Showa University Hospital. Data cleaning was applied to resolve defects of data, if necessary. We attempted to fill the template as completely as possible.

**Table 1:** Registration items in the HBOC registration system

Basic information for the subject or other family members
**1. Family relationship, 2.Gender, 3. Race**
4. Father’s individual number, 5. Mother’s individual number (number issued by this system)
**6. Date of birth** ^a^
7. Date of death, **8. Age at death, 9. Cause of death, 10. History of cancers**
**11. Menopausal state, 12. Menopausal age**, 13. Age at menarche, 14. Times of childbirth, 15. Age at first childbirth
Information on breast cancer^b^
**16. Onset age of breast cancer**
**17. Single or multiple, unilateral or bilateral, 18. Site (right or left), distant metastasis**
**19. Modality of diagnosis (trigger of finding a lesion)**
**20. Medical treatment (surgery, preoperative chemotherapy, post-operative chemotherapy, preoperative hormone therapy, post-operative hormone therapy, molecular-targeted drug, radiotherapy, no treatment)**
**21. Operation procedure, 22. Operation date**
**23. Histology, 24. Pathological tumor size (clinical tumor size), 25. Number of lymph node positive for cancer, 26. ER, 27. PGR, 28. HER2 (IHC), 29. HER2 (FISH)**
30. Ki67 score, 31. Epithelial marker, **32. Nuclear grade (NG)**
Information on ovarian cancer
**33. Onset age of ovarian cancer**
**34. Ovarian cancer, tubal cancer, peritoneal cancer**
**35. Histology, 36. Stage, 37. Degree of differentiation for cancer, 38. Metastatic sites**
*39. Date medical treatment was started, 40. Operation date, 41. Radicality*
*42. Date of detection of first recurrence*
Effect of chemotherapy for ovarian cancer
*43. Preoperative chemotherapy, 44. Regimen, 45. Best effect*
Post-operative chemotherapy^c^
*46. Regimen, 47. Period from completion of the last regimen to the recurrence, 48. Best effect, 49. Progression-free period*
Other cancers than breast and ovary (all cancers should be described if patients have a history of plural cancers)
**50. Type of other cancers, 51. Onset age**
**52. Other history associated with HBOC**
Information on genetic tests
**53. Date of genetic test for** ***BRCA***, **54.Type of examination, source of genetic test (company, medical institute, etc.), 55. Purpose of genetic test**
**56. Mutation of** ***BRCA1***, **Mutation site** ^**d**^ **(DNA and protein), 57. Mutation of** ***BRCA2***, **mutation site** ^**d**^ **(DNA and protein)**
Information on prophylactic surgery of the breast and the ovary
**58. Risk-reducing mastectomy (RRM), 59. Operation procedure for RRM, 60. Pathological findings of the resected specimen, 61. Execution age of RRM**
**62. Risk-reducing salpingo-oophorectomy (RRSO), 63. Operation procedure for RRSO, 64. Pathological findings of the resected specimen, 65. Execution age of RRSO**
Information on visiting a hospital
**66. First consultation at the hospital, 67. Final consultation at the hospital**

Bold characteristics indicate essential registration items for all subjects. Italic characteristics indicate essential registration items for BRCA mutation carriers. No. 1, 16, 33, 50, and 51 are essential registration items for registrees within the second relatives of the subject or uncles, who suffered from any cancers and registered by interview-based information (they are defined as the Others in Table 2)

^a^The date is deleted at submission (only month and year are registered) for protection of individual information

^b^We can register up to the third breast cancer, if patients had suffered from it

^c^We can register up to the third regimen, if patients had received one

^d^Based on the Myriad notation or HGVS notation

### Prevalence of *BRCA1/2* mutations

On the basis of the affected risk of *BRCA1/2* mutation, we prepared a prevalence table and compared the rate of each condition with that previously reported by Sugano et al. [6] As with the data for the USA, we referred to the report by Frank et al. [7] The nomenclature of the gene variants was based on both the Myriad notation (nucleotide number is described according to GenBank accession number U14680 for *BRCA1* and U43746 for *BRCA2*) and the HGVS standard notation [8].

### Tumor spectrum and clinical and pathological features of *BRCA*-related breast cancer

Based on the registered clinical data, we examined what types of cancer were frequently observed in Japanese *BRCA1/2* mutation carriers, and intrinsic subtypes of breast cancers in *BRCA1/2* mutation carriers based on ER, PgR, and HER2 status. Age of onset of cancer is one of the most important characteristics. We analyzed the difference in the onset age of breast cancer among the three groups using one-way analysis of variance, and results were considered statistically significant at *P* < 0.05. To analyze the difference in the onset age of ovarian cancer, we performed statistical analysis using the Student’s *t* test, and results were considered statistically significant at *P* < 0.05.

### Incidence of contralateral breast cancer

Regarding the incidence rate of contralateral breast cancer, we calculated the frequency of the onset of contralateral breast cancer using the person-year method. We defined the observation period as the term between the day on which a registree received *BRCA1/2* genetic testing and the latest day that we confirmed her condition at each institute. We calculated the incidence rate of breast cancer or ovarian cancer in unaffected *BRCA1/2* mutation carriers in the same manner.

### Risk-reducing surgery

The status of risk-reducing surgery, including risk-reducing mastectomy (RRM) and risk-reducing salpingo-oophrectomy (RRSO), is also presented. Clinical and pathological data were extracted from the registration database. We examined the frequency of secondary breast cancers and peritoneal cancers during the observation period and the frequency of occult cancers.

## Results

### Subjects and registration

Information on 846 families was collected from the four aforementioned medical institutions. We ascertained clinical and genetic characteristics of Japanese HBOC. Therefore, 13 foreign persons were excluded and 3 overlapping cases among two institutes were adjusted. Thus, we conducted the following analysis on 830 Japanese pedigrees. Among them, 944 individuals visited one of the four hospitals and provided informed consent for the registration project (22 males, 922 females, average age 46.9 years (20–85)) (Table 2). Therefore, we had 3867 registrees including 963 individuals who received *BRCA1/2* genetic testing (including those who underwent the testing outside of the four medical institutes). The deleterious *BRCA1/2* mutation was observed in 161 (19.7%) of 817 cases with no previous information on *BRCA1* and *BRCA2* mutation status (*BRCA1*: 84, *BRCA2*: 76, both: 1; 13 cases already had *BRCA1/2* genetic test results in their pedigree). Thus, these cases were excluded in the analysis of prevalence rate. In total, 240 *BRCA1* or *BRCA2* mutation carriers were registered in this study (*BRCA1*: 125, *BRCA2*: 113, both: 2). (Table 2).

**Table 2:** Characteristics of Japanese registrees

		Male	Female	Average age at BRCA1/2 genetic test (youngest–oldest)
Pedigree	830			
Registrees^a^	3867	1176	2691	
Subjects^b^	944	22	922	46.9 (20–85)
Others^c^	2923	1154	1769	
Clients who received BRCA1/2 genetic testing	963	23	940	47.0 (20–85)
*BRCA1* mutation positive	127	5	122	45.0 (23–79)
*BRCA2* mutation positive	115	4	111	45.4 (23–74)

^a^Registrees consisted of subjects and the others

^b^Clients who provided informed consent at the time of registration in any of the four participating medical institutes

^c^Registrees within the second relatives of the subjects or uncles, who suffered from any cancers and registered by interview-based information

### Prevalence of *BRCA1/2* mutations

Pathological mutations of *BRCA1* and *BRCA2* were widely distributed across both whole genes (Table 3). Most deleterious mutations had been reported twice or less at the four institutions, but the L63X mutation in exon 5 of *BRCA1* was frequently encountered, and it thus might be a mutation hotspot in Japan. Fifty-three variants of uncertain significance (VUS) were detected in 6.5% of all genetic tests for *BRCA1/2* (*BRCA1*: 21, *BRCA2*: 31, both: 1) (Table 4). In addition, there were eight VUS having simultaneous deleterious mutations in *BRCA1/2*.

**Table 3:** Deleterious mutations of *BRCA1* and *BRCA2* in the Japanese population

Base pair change			Amino-acid change	Times	Base pair change			Amino-acid change	Times
Myriad		HGVS	HGVS		Myriad		HGVS	HGVS	
*BRCA1*									
	307T>A	c.188T>A	p.Leu63Ter	26		3699ins4	c.3580_3581 insACCC	p. Thr1194AsnfsThr26	1
	2919C>T	c.2800C>T	p.Gln934Ter	6		4184del4	c.4065_4068 delTCAA	p.Asn1355LysfsTer10	1
	2508delGA	c.2389_2390delGA	p.Glu797ThrfsTer3	4		4457ins4	c.4338_4339 insTCAA	p. Gln1447SerfsTer16	1
	3561delG	c.3442delG	p.Glu1148ArgfsTer7	4		5291insA	c.5172dupA	p.Glu1725ArgfsTer7	1
	297C>T	c.178C>T	p.Gln60Ter	2		5589del8	c.5470_5477 delATTGGGCA	p.Ile1824AspfsTer3	1
	309T>C	c.190T>C	p.Cys64Arg	2		546G>T	c.427G>T	p.Glu143Ter	1
	3759G>T	c.3640G>T	p.Glu1214Ter	2		1173G>T	c.1054G>T	p.Glu352Ter	1
^a^	5083C>T	c.4964C>T	p.Ser1655Phe	2		1740C>T	c.1621C>T	p.Gln541Ter	1
	575delCA	c.456_457delCA	p.Ser153CysfsTer5	2		190G>A	c.71G>A	p.Cys24Tyr	1
	Exon1a–Exon2del	c.(?_-1387-1)_ (80+1_81-1)del		2		2178C>T	c.2059C>T	p.Gln687Ter	1
	IVS20-1G>C	c.5278-1G>C		2		2841G>T	c.2722G>T	p.Glu908Ter	1
	185insA	c.66dupA	p. Glu23ArgfsThr18	1		3471C>T	c.3352C>T	p.Gln1118Ter	1
	185delAG	c.68_69delAG	p.Glu23ValfsThr17	1		360C>T	c.241C>T	p.Gln81Ter	1
	589delCT	c.470_471delCT	p.Ser157Terfs	1		3992T>A	c.3873T>A	p.Cys1291Ter	1
	604delTG	c.485_486delTG	p.Val162GlufsTer19	1		5622C>T	c.5503C>T	p.Arg1835Ter	1
	819delA	c.700delA	p.Thr234GlnfsTer13	1	^a^	5264C>A	c.5145C>A	p.Ser1715Arg	1
	1158delCT	c.1039_1040delCT	p.Leu347ValfsTer2	1		EXON1a-9del	c.(?_-1387-1)_ (593+1_594-1)del		1
	1343delA	c.1224delA	p.Val409Ter	1		EXON3del	c.(80+1_81-1)_ (134+1_135-1)del		1
	1406insA	c.1287dupA	p.Asp430ArgfsTer6	1		EXON8del	c.(441+1_442-1)_ (546+1_547-1)del		1
	1623del5	c.1504_1508 delTTAAA	p.Leu502AlafsTer2	1		EXON20del	c.(5193+1_5194-1)_(5277+1_5278-1) del		1
	2632delA	c.2513delA	p.Asn838ThrfsTer8	1	^a^	IVS4-2A>C	c.135-2A>C		1
	2730delCC	c.2611_2612delCC	p.Pro871ValfsTer31	1	^a^	IVS4-2A>G	c.135-2A>G		1
	2805delA	c.2686delA	p.Ser896Valfs104	1		IVS17+3A>G	c.5074+3A>G		1
*BRCA2*									
	5804del4	c.5576_5579 delTTAA	p.Ile1859LysfsTer3	8		6674del**5**	c.6446_6450 delTTAAA	p.Ile2149SerfsTer25	1
	1506delA	c.1278delA	p.Asp427ThrfsTer3	5		6696delTC	c.6468_6469delTC	p.Gln2157IlefsTer18	1
	8732C>A	c.8504C>A	p.Ser2835Ter	4		6744delA	c.6516delA	p.Val2174LeufsTer17	1
	9345G>A	r.[8954_9117del] c.9117G>A	p.Val2985GlyfsTer4 p.Pro3039=	4		6848del4	c.6620_6623 delCAAA	p.Thr2207IlefsTer2	1
	7180C>T	c.6952C>T	p.Arg2318Ter	3		6854delTA	c.6626_6627delTA	p.Ile2209ArgfsTer15	1
	8817insA	c.8589dupA	p.Ala2864SerfsTer5	3		7004del4	c.6776_6779 delATGA	p.Asn2259ArgfsTer20	1
	8857G>T	c.8629G>T	p.Glu2877Ter	2		7661insT	c.7433dupT	p.Leu2478PhefsTer19	1
	9304C>T	c.9076C>T	p.Gln3026Ter	2		7865insT	c.7638dupT	p.Lys2547Ter	1
	3423del4	c.3195_3198 delTAAT	p.Asn1066LeufsTer10	2		1627A>T	c.1399A>T	p.Lys467Ter	1
	3463delT	c.3235delT	p.Ser1079LeufsTer8	2		4123G>T	c.3895G>T	p.Glu1299Ter	1
	983del4	c.755_758 delACAG	p.Asp252ValfsTer24	1		4250C>A	c.4022C>A	p.Ser1341Ter	1
	1033insA	c.805dupA	p. Thr269AsnfsTer7	1		4917G>A	c.4689G>A	p.Trp1563Ter	1
	1524delGA	c.1296_1297 delGA	p.Asn433GlnfsTer18	1		6369T>G	c.6141T>G	p.Tyr2047Ter	1
	1866del13	c.1638_1650del13	p. Cys546TrpfsTer8	1		6856G>T	c.6628G>T	p.Glu2210Ter	1
	2041delA	c.1813delA	p.Ile605TyrfsTer9	1		7708C>T	c.7480C>T	p.Arg2494Ter	1
	2041insA	c.1813dupA	p.Ile605AsnfsTer11	1		7786C>T	c.7558C>T	p.Arg2520Ter	1
	3036del4	c.2808_2811 delACAA	p.Ala938ProfsTer21	1		5873C>A	c.5645C>A	p.Ser1882Ter	1
	3292delC	c.3064delC	p.His1022IlefsTer22	1		9337C>T	c.9109C>T	p.Gln3037Ter	1
	3827delGT	c.3599_3600delGT	p.Cys1200Terfs	1		9610C>T	c.9382C>T	p.Arg3128Ter	1
	4692delCA	c.4464_4465delCA	p.His1488GlnfsTer25	1	^a^	319T>A	c.91T>A	p.Trp31Arg	1
	4796delG	c.4568delG	p.Gly1523ValfsTer20	1		7235G>A	c.7007G>A	p.Arg2336His	1
	5358del4	c.5130_5133 delTGTA	p.Tyr1710Terfs	1	^a^	8251A>G	c.8023A>G	p.Ile2675Val	1
	5903delG	c.5675delG	p.Gly1892ValfsTer17	1		9382C>T	c.9154C>T	p.Arg3052Trp	1
	5999del4	c.5771_5774 delTTCA	p.Ile1924ArgfsTer38	1		IVS15+1G>T	c.7601+1G>T		1
	6386delC	c.6158delC	p.Ser2053LeufsTer17	1	^a^	IVS5+1G>A	c.475+1G>A		1
	6673delAT	c.6445_6446delAT	p.Ile2149Terfs	1	^a^	IVS8-1G>T	c.682-1G>T		1

Times: reported frequency

^a^Suspected deleterious

**Table 4:** Variants of uncertain significance (VUS) of *BRCA1* and *BRCA2* in the Japanese population

Base pair change			Amino-acid change	Times	Base pair change			Amino-acid change	Times
Myriad		HGVS	HGVS		Myriad		HGVS	HGVS	
*BRCA1*									
^a^	273C>T	c.154C>T	p.Leu52Phe	3		2683A>C	c.2564A>C	p.Gln855Pro	1
	745C>T	c.626C>T	p.Pro209Leu	3	^a^	2845A>T	c.2726A>T	p.Asn909Ile	1
	1321G>A	c.1202G>A	p.Gly401Glu	2		3568C>G	c.3449C>G	p.Pro1150Arg	1
	5677A>G	c.5558A>G	p.Tyr1853Cys	2		4129A>G	c.4010A>G	p.Asp1337Gly	1
^a^	517G>A	c.398G>A	p.Arg133His	1		4253C>T	c.4134C>T	p.Val1378Val	1
	Unknown	Unknown	p.Arg166Ser	1		4879C>T	c.4760C>T	p.Ser1587Leu	1
	895A>T	c.776A>T	p.Glu259Val	1		5054G>C	c.4935G>C	p.Arg1645Ser	1
	1476G>C	c.1357G>C	p.Glu453Gln	1	^a^	5466A>C	c.5347A>C	p.Met1783Leu	1
^a^	1998G>A	c.1879G>A	p.Val627Ile	1		IVS17-9A>G	c.5075-9A>G		1
	2646A>G	c.2527A>G	p.Thr843Ala	1					
*BRCA2*									
^a^	281G>A	c.53G>A	p.Arg18His	4		6257T>G	c.6029T>G	p.Val2010Gly	1
^a^	6197A>C	c.5969A>C	p.Asp1990Ala	4		7777A>G	c.7549A>G	p.Thr2517Ala	1
^a^	779T>C	c.551T>C	p.Leu184Pro	3		7910A>T	c.7682A>T	p.Gln2561Leu	1
^a^	IVS3-10A>G	c.317-10A>G		3		8633C>T	c.8405C>T	p.Pro2802Leu	1
	851T>G	c.623T>G	p.Val208Gly	2		8719A>T	c.8491A>T	p.Met2831Leu	1
	5082T>A	c.4854T>A	p.Asp1618Glu	2		8927A>G	c.8699A>G	p.Asp2900Gly	1
	324T>G	c.96T>G	p.Phe32Leu	1		9394C>T	c.9166C>T	p.His3056Tyr	1
	478C>G	c.250C>G	p.Gln84Glu	1		10,049T>G	c.9821T>G	p.Leu3274Trp	1
	2978T>C	c.2750T>C	p.Val917Ala	1	^a^	661del3	c.433_435delGTT	p.Val145del	1
	3648T>A	c.3420T>A	p.Ser1140Arg	1		IVS5+9A>T	c.475+9A>T		1
	4655A>G	c.4427A>G	p.Asp1476Gly	1		IVS6-12C>A	c.517-12C>A		1
	5410G>A	c.5182G>A	p.Asp1728Asn	1		IVS6-2A>G	c.517-2A>G		1
	5545G>C	c.5317G>C	p.Glu1773Gln	1		IVS11-202 del20	c.6842-202_ 6842-183del		1

Times: reported frequency

^a^Favor polymorphism

A prevalence table was generated based on the data from this trial registration. In most groups, except the male breast cancer group, the *BRCA1/2* mutation frequency was higher in Japanese individuals than that in Americans (non-Ashkenazi Jews) (Table 5).

**Table 5:** Prevalence of deleterious mutations in *BRCA1* and *BRCA2* based on this study

Family history of breast cancer or ovarian cancer	Among first- and second-degree relatives				No family history of breast or ovarian cancer	Family history in only third-degree relatives
Breast cancer <50 y	−	+	−	+		
Ovarian cancer (at any age)	−	−	+	+		
**Patient’s history**						
Breast cancer ≥ 50 y	2/58	9/58	6/24	1/8	0/26	0/8
	3.4%	15.5%	25.0%	12.5%	0%	0%
Breast cancer < 50 y	21/125	45/139	27/61	17/27	16/164	1/17
	16.8%	32.4%	44.3%	63.0%	9.8%	5.9%
Ovarian cancer with no breast cancer at any age	0/2	1/1	1/2	−	1/5	0/1
	0%	100%	50.0%	−	20.0%	0%
Breast cancer and ovarian cancer at any age	1/1	1/2	4/4	−	1/6	−
	100%	50%	100%	−	16.7%	−
Male breast cancer at any age	0/1	0/1	−	−	0/1	−
	0%	0%	−	−	0%	−
No breast cancer or ovarian cancer at any age	0/16	2/27	3/20	0/7	1/4	0/1
	0%	7.4%	15.0%	0%	25.0%	0%

### Tumor spectrum and clinical and pathological features of *BRCA*-related breast cancer

We examined what type of cancer occurred in *BRCA1/2* mutation carriers. As expected, breast cancer was the most frequent followed by ovarian cancer and uterine cancer. Of 127 *BRCA1* mutation carriers, 94 had a history of or were currently suffering from breast cancer (67 single cases, 24 cases with 2 simultaneously metachronous breast cancers, 3 with 3 simultaneous or metachronous cancers). Of 115 *BRCA2* mutation carriers, 98 subjects had breast cancer (78 single cases, 18 cases with 2 breast cancers, 2 with 3 breast cancers). Ovarian cancers were observed in 14 cases (*BRCA1*; 9, *BRCA2*; 5). However, other cancers except endometrial cancer and cervical cancer were only reported once.

We examined intrinsic subtypes of breast cancer in *BRCA1/2* mutation carriers based on the information about their ER, PgR, and HER2 status. Of 66 breast cancers in *BRCA1* mutation carriers, 75.8% were triple-negative breast cancer (TNBC) and 21.2% were of the luminal type. Whereas of 59 breast cancers in *BRCA2* mutation carriers, 18.6% were TNBC and 64.4% were luminal type, respectively.

The distribution of onset age for breast cancer and ovarian cancer was compared among *BRCA1/2*-positive and *BRCA1/2*-negative groups. For first breast cancers, the average age of onset in the *BRCA1*- and *BRCA2*-positive groups (90 and 98 cases) and BRCA-negative group (588 cases) was 40.2 years (95% confidence interval (CI) 38.15–42.25), 41.7 years (95% CI 39.71–43.70), and 45.4 years (95% CI 44.54–46.26), respectively. The onset age of breast cancer in the *BRCA1*-positive group was significantly lower than that of the *BRCA*-negative group (*P* < 0.001). A significant difference was also observed between the *BRCA2*-positive group and *BRCA*-negative group (*P* = 0.004). The difference regarding onset age of breast cancer between the *BRCA1*- and *BRCA2*-group was not statistically significant. For ovarian cancer, the average age of onset was 51.6 years (SD: 9.90) in the *BRCA1/2* mutation-positive group and 44.5 years (SD: 10.38) in the mutation-negative group, but the difference in the onset age of ovarian cancer was not significant (*P* = 0.072); however, the onset age of ovarian cancer in the *BRCA1/2*-positive group tended to be higher than that in the *BRCA*-negative group. The number of cases with ovarian cancer was too small for a valid statistical analysis (14 and 15 cases in *BRCA1/2* mutation-positive and -negative group, respectively).

The reason to receive genetic testing for BRCA1/2 was also registered. Personal healthcare was the most frequent answer, followed by facilitation of decision-making for an operation. Indeed, out of 50 mutation-positive cases, only 6 cases (12.0%) chose breast conserving surgery, whereas of 218 mutation-negative cases, 120 cases (55.0%) received breast conserving surgery.

### Incidence of contralateral breast cancer

We also calculated the incidence rate of contralateral breast cancer development in patients with unilateral breast cancer. The average observation periods after the genetic tests were 27.3 and 16.2 months for *BRCA1/2* mutation-positive and mutation-negative groups, respectively. Three contralateral breast cancers occurred in *BRCA1/2* mutation carriers (3/302.6 person-years), and the annual incidence rate was 0.99%. Breast cancer occurrence was observed in two *BRCA1/2* non-mutation carriers (2/650.7 person-years, 0.33%/year). The incidence of cancer in 37 unaffected *BRCA1/2* mutation carriers was also examined. The mean observation period after genetic testing was 17.2 months (1–69 months). The average age at the time of genetic testing was 40.1 years (23–64 years) and, during the observation period, two incidences of breast cancer occurred (2/53.0 person-years). The incidence of breast cancer in unaffected *BRCA1/2* mutation carriers was 3.8%/year. The onset of ovarian cancer was not detected during this observation period in the same unaffected carriers.

### Risk-reducing surgery

RRM and RRSO were performed in 26 and 62 patients, respectively, among 240 mutation carriers. All 26 RRM cases were classified as contralateral RRM. The average age at the RRM was 43.8 years (33–56 years). The average period from breast cancer onset to RRM was 2.46 years on average (0–14 years). Occult cancer was detected in two patients by post-operative pathological examination of RRM (7.7%). Metachronous breast cancer from residual breast tissue was not detected during the observation period after RRM (mean observation period: 18.6 months (2–43.2 months)).

The average age at RRSO was 49.2 years (range, 36–65 years), and the mean interval from the onset of breast cancer was 5.1 years (except in five individuals without breast cancer). Two occult cancers (one fallopian tube cancer and one ovarian cancer) were detected upon pathological examination of the resected specimen (3.2%). Peritoneal cancers were not observed during the follow-up period after RRSO (average observation period: 21.7 months (0–50.8 months)).

## Discussion

Until recently, the actual state of clinical practice for HBOC in our country remained unclear. Information is not available on how risk-reducing surgeries are performed under the present medical insurance system in Japan or on how many cases of risk-reducing surgeries are performed. Our aim with this registration system is to clarify pathological and clinical features of HBOC among Japanese and to link authorized systems of medical institutes to improve awareness of HBOC in the future.

One of the merits of this registration system is that registrees, who received genetic testing for *BRCA1/2*, including their family, can be followed up by annual update. The annual update of registration data enables us to perform prospective analysis and to improve data quality, providing revised clinical data. We collected HBOC data from four institutions that most actively work on HBOC clinical practice and perform extensive *BRCA1/2* genetic tests in Japan. No data on *BRCA* mutation carriers compared to the mutation-negative population are available in Japan at such a large scale. Approximately 40% or more of annual genetic *BRCA1/2* tests are ordered by these four institutions in Japan. Therefore, this trial registration would aptly reflect the characteristics of *BRCA1/2* mutations in the Japanese HBOC.

Nakamura et al. [5] showed deleterious mutations in 30.7% of patients with breast cancer and confirmed their clinical criterion for high risk. In the present registration, pathological mutations were confirmed in 19.7% of the subjects who first underwent genetic testing for *BRCA1/2* in their pedigrees. This lower prevalence rate might be explained as follows: first, in Nakamura’s study, almost all subjects had personal history and family history of breast cancer and were more likely to be HBOC. In this registration, all subjects who received BRCA genetic testing were enrolled regardless of their personal history and family history. Second, even in Japan, as of late, genetic testing has become popular and widespread. One of the reasons is the so called “Angelina effect” [9]. Even patients without high risk of developing breast cancer might undergo genetic testing. Third, subjects who were enrolled in clinical examinations on PARP inhibitors were also included (8.3% of all subjects). They were not always likely to be *BRCA1/2* mutation carriers based on clinical characteristics and family history.

Mutations were distributed across almost all regions of both genes. Mutation frequency was particularly high for L63X in *BRCA1*, and this nonsense mutation seemed to be a hotspot in the Japanese population. Based on earlier studies, this mutation is a founder mutation by haplotype analysis, and carriers with the mutation are mainly from the eastern part of Japan [10, 11].

In the prevalence table, Japanese individuals had a higher mutation-positive rate for *BRCA1/2* than Americans (except for Ashkenazi Jews) in almost all groups except for the male breast carcinoma groups. Sugano et al. [6] showed that the relative risk of *BRCA1/2* mutation in Japanese individuals is significantly higher than that in Americans (barring Ashkenazi Jews) (odds ratio 1.87, *P* = 0.005). The current study included more subjects and provided more reasonable and realistic results than the previous study. The mutation prevalence of *BRCA1/2* mutation carriers in Japanese individuals is higher than that in the USA except for the groups with male breast cancer [7]. Furthermore, our results seem to be more reasonable than the previous data. The prevalence rate of probands with breast cancer over 50 years of age, with no family history of early onset breast cancer and ovarian cancer, was 3.4% in our study and 2.3% in non-Ashkenazi Americans, whereas it was 21.4% in the previous study [6, 7]. Compared with the prevalence rate in the German consortium of HBOC, the overall prevalence rate was similar to that in Germany. In the report, high mutation frequency was observed in families with two or more ovarian cancers (41.9%), and families with one or more breast cancer and one ovarian cancer (41.6%) [12]. According to our data, the prevalence rate of Japanese probands with early onset breast cancer and family history of ovarian cancer is also over 40%. Family history of ovarian cancer is an important factor among studies in each country.

The frequency of TNBC is considered to be 10–15% among breast cancer subtypes [13]. The ratio of TNBC in individuals positive for *BRCA1* mutations was 75.8% in this study. According to many reports, the frequency of TNBC in *BRCA1* mutation carriers is ~60–80% [14]. CIMBA reported the frequency to be 68% [15].

The reason for receiving *BRCA1/2* genetic testing was also registered. The most common answer is that subjects received genetic testing for their own healthcare. Many of the mutation carriers received medical intervention such as regular medical checkups or risk-reducing surgeries under cooperation among the departments of breast surgery, gynecology, and clinical genetics.

The onset age of breast cancer in the *BRCA1 or BRCA2* mutation-positive group was significantly lower than that in the *BRCA* mutation-negative group (40.2 years, 41.7 years, and 45.4 years, respectively). This is also ~20 years younger than that in the Japanese general population (60.8 years) [16, 17]. Meanwhile, the average age of onset for ovarian cancer in the mutation-positive group, which is critical information for the appropriate age for RRSO, was approximately 7 years older than that of the mutation-negative group. However, this was still lower than that of the Japanese general population (61.1 years) [16, 17]. The tendency of older age of onset for ovarian cancer in *BRCA1/2* mutation carriers could not be confirmed statistically because the number of cases with ovarian cancer was too low. National Comprehensive Cancer Network guidelines propose that *BRCA1/2* mutation carriers undergo RRSO at the age of 35–40 years [18]. The reasonable age for RRSO in Japan might be older than the age recommended by NCCN. RRSO is also recommended in the Japanese Breast Cancer Practice Guidelines. The appropriate age for RRSO should be considered in Japan as well [19].

The incidence rate of contralateral breast cancer in a *BRCA1/2* mutation carrier was 0.99%/year, which was three times higher than that for a person without the mutation. However, the risk seems lower than that reported in previous foreign studies. Brekelmans et al. [20] reported that the risk of metachronous contralateral breast cancer was 3.1%/year in both *BRCA1* and *BRCA2* mutation carriers. A meta-analysis of cumulative risks for contralateral breast cancer in *BRCA1/2* mutation carriers with a first breast cancer showed a cumulative 10-year incidence rate of 27% and 19% for contralateral breast cancer in *BRCA1* and *BRCA2* mutation carriers, respectively [21]. Therefore, further long-term and large-scale studies are required. In unaffected *BRCA1/2* mutation carriers, two breast cancers, which occurred in patients in their 40s, were detected during the observation period of 17.2 months. The incidence rate of breast cancer is 3.8%/year in unaffected *BRCA1/2* mutation carriers.

Preventive surgeries are not yet widespread in Japan; RRM and RRSO were performed in 26 and 62 *BRCA1/2* mutation carriers, respectively, at the four institutions studied. According to a recent report from overseas, preventive surgeries are being performed very frequently; 46% of *BRCA1/2* mutation carriers underwent RRM and 70–80% of *BRCA1/2* mutation carriers underwent RRSO [22]. However, a report indicated that RRSO is performed in 52.4% of cases in Korea, showing a slightly lower frequency compared to that in Europe or the USA [23]. In comparison to these countries, only a few people undergo preventive surgeries in Japan. The frequency of occult cancer in the resected specimens was 7.7% in RRM and 3.2% in RRSO. Evans et al. reported that occult cancers occurred in 2.3% of RRM cases, whereas reports from the USA and Korea showed that the incidence of occult cancers in RRSO is 5.4% and 11.7%, respectively. Our data indicate a relatively low incidence despite the detailed pathological examination of resected ovaries and fallopian tubes. However, the sample size was small. Therefore, further studies are required to confirm these numbers [24, 25].

This study of the trial registration system to estimate clinical features of patients with HBOC presents some limitations. First, the medical staff who inputted data were largely specialized in breast cancer. Therefore, information about ovarian cancer was frequently lacking, particularly information about the regimen for chemotherapy and its effects, despite data cleaning. Additionally, we did not have sufficient data on ovarian cancers in *BRCA* mutation carriers. Furthermore, these data were collected from only four institutes and unevenly distributed within Tokyo or the greater Tokyo area. Therefore, the results do not reflect characteristics of the entire population of Japanese *BRCA* mutation carriers, although over 40% of clients receiving *BRCA1/2* genetic testing were covered in these four institutes.

Despite these limitations, we recognized the outline of clinical features of *BRCA1/2* mutation carriers and clinical characteristics of breast and ovarian cancers with HBOC, and this trial registration elucidated some issues that should be addressed in the registration system described above.

In conclusion, we established a nationwide registration system for subjects receiving genetic testing for *BRCA1/2* and started the official registration in March 2016. The registration system provides annual updated data reflecting the actual conditions for not only the subjects, but also relatives with cancer, an advantage of the Japanese registration system. In the future, it will be necessary to collect additional HBOC cases and to follow them up for a long period to clarify the genetic and clinical characteristics of HBOC in Japan.

## Electronic supplementary material

Supplementary Figure 1
