# Supplementary figures and images for: Genetic and clinical characteristics in Japanese hereditary breast and ovarian cancer: first report after establishment of HBOC registration system in Japan
Source: J Hum Genet. 2017 Nov 8;63(4):447–57. doi: 10.1038/s10038-017-0355-1 (PMC8716335; doi:10.1038/s10038-017-0355-1)

Supplementary Figure 1.


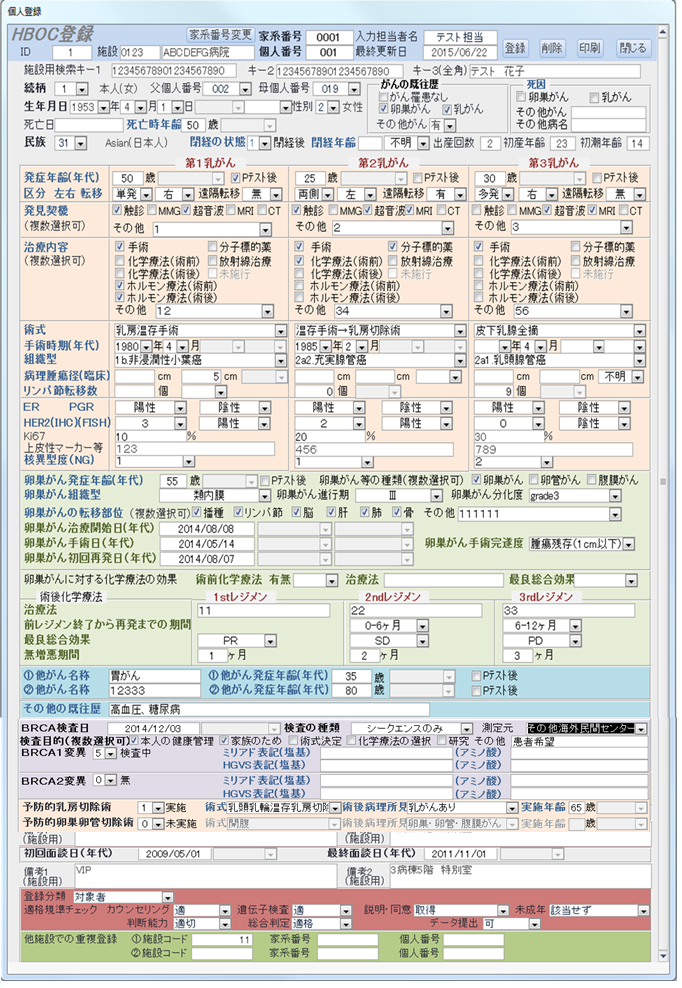

Supplement: Supplementary file 1 — Supplementary Figure 1 [file 10038_2017_355_MOESM1_ESM.docx]
